# Efficacy and safety of tepotinib in Asian patients with advanced NSCLC with *MET* exon 14 skipping enrolled in VISION

**DOI:** 10.1038/s41416-024-02615-9

**Published:** 2024-04-04

**Authors:** Terufumi Kato, James Chih-Hsin Yang, Myung-Ju Ahn, Hiroshi Sakai, Masahiro Morise, Yuh-Min Chen, Ji-Youn Han, Jin-Ji Yang, Jun Zhao, Te-Chun Hsia, Karin Berghoff, Rolf Bruns, Helene Vioix, Simone Lang, Andreas Johne, Xiuning Le, Paul K. Paik

**Affiliations:** 1 Department of Thoracic Oncology, Kanagawa Cancer Center, Yokohama, Japan; 2 Department of Medical Oncology, National Taiwan University Cancer Center, Taipei, Taiwan; 3 Division of Hematology Oncology, Department of Medicine, Samsung Medical Center, Sungkyunkwan University School of Medicine, Seoul, Republic of Korea; 4 Department of Thoracic Oncology, Saitama Cancer Center, Kitaadachi-gun, Japan; 5 Department of Respiratory Medicine, Nagoya University Graduate School of Medicine, Nagoya, Japan; 6 Department of Chest Medicine, Taipei Veterans General Hospital, and School of Medicine, National Yang-Ming University, Taipei, Taiwan; 7 The Center for Lung Cancer, National Cancer Center, Goyang, Republic of Korea; 8 Department of Oncology, Guangdong Lung Cancer Institute, Guangdong General Hospital and Guangdong Academy of Medical Sciences, Guangzhou, China; 9 Department of Thoracic Oncology, Peking University Cancer Hospital and Institute, Beijing, China; 10 Department of Internal Medicine, China Medical University Hospital, Taichung, Taiwan; 11 Global Patient Safety, Merck Healthcare KGaA, Darmstadt, Germany; 12 Department of Biostatistics, Merck Healthcare KGaA, Darmstadt, Germany; 13 Global Evidence and Value Department, Merck Healthcare KGaA, Darmstadt, Germany; 14 Global Clinical Development, Merck Healthcare KGaA, Darmstadt, Germany; 15 Department of Thoracic Head and Neck Medical Oncology, The University of Texas MD Anderson Cancer Center, Houston, TX USA; 16 Department of Medicine, Weill Cornell Medical College, New York, NY USA; 17 Department of Medicine, Thoracic Oncology Service, Memorial Sloan-Kettering Cancer Center, New York, NY USA; 18 Present Address: Department of Thoracic Oncology, Ageo Central General Hospital, Saitama, Japan

**Keywords:** Non-small-cell lung cancer, Non-small-cell lung cancer

## Abstract

**Background:**

Tepotinib, a MET inhibitor approved for the treatment of *MET* exon 14 (*MET*ex14) skipping NSCLC, demonstrated durable clinical activity in VISION (Cohort A + C; N = 313): objective response rate (ORR) 51.4% (95% CI: 45.8, 57.1); median duration of response (mDOR) 18.0 months (95% CI: 12.4, 46.4). We report outcomes in Asian patients from VISION (Cohort A + C) (cut-off: November 20, 2022).

**Methods:**

Patients with advanced *MET*ex14 skipping NSCLC, detected by liquid or tissue biopsy, received tepotinib 500 mg (450 mg active moiety) once daily. Primary endpoint: objective response (RECIST 1.1) by independent review. Secondary endpoints included: DOR, progression-free survival (PFS), overall survival (OS), safety, and health-related quality of life (HRQoL).

**Results:**

Across treatment lines in 106 Asian patients (39.6% female, 43.4% smoking history, 79.2% adenocarcinoma, 47.2% treatment-naive), ORR was 56.6% (95% CI: 46.6, 66.2), mDOR 18.5 months (10.4, ne), mPFS 13.8 months (10.8, 22.0), and mOS 25.5 months (19.3, 36.4). Consistent efficacy observed, regardless of baseline characteristics. HRQoL remained stable during treatment. Treatment-related adverse events (TRAEs) occurred in 95.3% of patients (39.6% Grade ≥3). Most common TRAEs: peripheral edema (62.3%), creatinine increase (38.7%).

**Conclusions:**

Tepotinib demonstrated robust and durable efficacy, with a manageable safety profile, in Asian patients with *MET*ex14 skipping NSCLC.

**Clinical trial registration:**

NCT02864992

## Background

Mesenchymal–epithelial transition exon 14 (*MET*ex14) skipping is a *MET* alteration that occurs in 3–4% of patients with non-small cell lung cancer (NSCLC), including 1–4% of Asian patients with lung adenocarcinoma [1–7]. *MET*ex14 skipping can be detected in tumor tissue biopsy (TBx) as well as in liquid biopsy (LBx; plasma circulating tumor DNA [ctDNA]), which are complementary approaches for the identification of actionable gene alterations in NSCLC [8].

Increasing evidence suggests that tumors harboring such mutations are sensitive to MET inhibition, and a number of selective MET tyrosine kinase inhibitors have demonstrated clinical activity in patients with *MET*ex14 skipping NSCLC [2]. Amongst them, tepotinib (Tepmetko^®^, Merck Healthcare KGaA, Darmstadt, Germany), an oral, once-daily, highly selective, potent MET inhibitor has shown clinical activity in MET-driven tumors [9, 10]. Tepotinib is currently approved for treating advanced or metastatic *MET*ex14 skipping NSCLC in many countries in Europe, North America, South America, and Asia, including Hong Kong, Japan, Singapore, South Korea, Taiwan, India, and Macao, and is the first MET inhibitor with full approval in China.

VISION (NCT02864992) is a Phase II study of tepotinib that enrolled patients based on TBx and/or LBx detection of *MET*ex14 skipping advanced/metastatic NSCLC (Cohorts A and C) [10, 11]. In the global population of the combined VISION Cohort A + C (N = 313), tepotinib demonstrated robust and durable clinical activity in patients with *MET*ex14 skipping NSCLC in long-term follow-up (median 32.6 months [range: 0.3–71.9]; data cut-off: November 20, 2022), with an objective response rate (ORR) of 51.4% (95% confidence interval [CI]: 45.8, 57.1), median duration of response (DOR) of 18.0 months (95% CI: 12.4, 46.4), and median progression-free survival (PFS) of 11.2 months (95% CI: 9.5, 13.8) [12].

Differences in the epidemiology, clinicopathologic characteristics, and prognosis of NSCLC have been reported between Asian and non-Asian populations [13]. Furthermore, the efficacy and safety of anticancer drugs have the potential to vary between patients from different ethnic groups, for example due to genetic or environmental factors, or regional differences in practice patterns [14]. To evaluate the efficacy and safety of tepotinib in patients of Asian ethnicity, we report outcomes in the subgroup of Asian patients enrolled in the VISION study (Cohort A + C), including health-related quality of life (HRQoL).

## Methods

The full methodology of the VISION study has been published previously and the protocol is available online [10, 12].

### Study design

VISION is a Phase II, single-arm, open-label, multicenter study of tepotinib in patients with NSCLC harboring *MET*ex14 skipping (Cohorts A and C). Cohort C (>18 months’ follow-up) is an independent cohort, designed to confirm findings from Cohort A (>35 months’ follow-up). Data cut-off was November 20, 2022. Patients received 500 mg (450 mg active moiety) tepotinib once daily. The treatment continued until disease progression, consent withdrawal, or adverse events (AEs) leading to discontinuation.

### Patients

Patients were ≥18 years of age with histologically or cytologically confirmed locally advanced or metastatic NSCLC (all types including squamous and sarcomatoid) harboring *MET*ex14 skipping, detected by TBx and/or LBx. Eligible patients had measurable disease according to the Response Evaluation Criteria in Solid Tumors (RECIST) v1.1, an Eastern Cooperative Oncology Group performance status (ECOG PS) of 0 or 1, and no epidermal growth factor receptor (*EGFR)* mutations or anaplastic lymphoma kinase (*ALK)* rearrangements. Patients who had received up to two lines of prior therapy for advanced or metastatic NSCLC were eligible. Prior immunotherapy was permitted, however prior use of MET inhibitors was not allowed.

Patients had to test positive for *MET*ex14 skipping in either ctDNA isolated from a fresh plasma sample (LBx) or RNA isolated from fresh or archival tumor tissue (TBx). During prescreening, central next-generation sequencing using the Oncomine^TM^ Focus Assay (52 genes, Thermo Fisher Scientific, Waltham, MA, USA) or the Archer^®^MET companion diagnostic assay (ArcherDx, Boulder, CO, USA) was carried out to analyze TBx, and the Guardant360^®^ assay (73 genes, Guardant Health, Redwood City, CA, USA) or the Archer^®^MET diagnostic assay were used for LBx. Patients in Japan could enroll without prescreening based on local *MET*ex14 skipping detection in TBx via a real-time polymerase chain reaction assay as part of a nationwide cancer genomic screening project (LC-SCRUM).

### Study endpoints and assessments

The primary endpoint was a confirmed objective response (defined as a complete or partial response [PR]) by independent review committee (IRC) using RECIST v1.1. Secondary endpoints included DOR, PFS, overall survival (OS), safety, and HRQoL. AEs were assessed by investigator using the National Cancer Institute Common Terminology Criteria for Adverse Events, v4.03. Patient-reported outcomes were assessed with the use of the European Organization for Research and Treatment of Cancer (EORTC) Quality of Life Questionnaire Lung Cancer Modules 13 and 30 (EORTC QLQ-LC13 and EORTC QLQ-C30), and the European Quality of Life five-dimension five-level questionnaire (EQ-5D-5L). Global health status (GHS) and the following symptoms were derived from EORTC QLQ-LC13: cough (items 1 and 2), dyspnea (items 3, 4, and 5), and chest pain (item 10). Linear mixed model regression was performed to obtain the mean change from baseline for each of the patient-reported outcomes (PROs); an increase or decrease of >10 points was considered to be clinically meaningful.

### Statistical analysis

No formal statistical comparisons were conducted; all statistical analyses used descriptive summary statistics. Predefined analysis sets for all endpoints included *MET*ex14 skipping detection by TBx, LBx, and the combined group (either biopsy method; TBx and/or LBx) [10]. Kaplan–Meier methods were used to analyse DOR, PFS, and OS. Qualitative variables and rates were summarized by counts and percentages along with 2-sided exact Clopper-Pearson 95% CIs. The safety population included all the patients who had enrolled in the study and received at least one dose of tepotinib.

## Results

### Patients

At the data cut-off for this analysis (November 20, 2022), 2118 Asian patients were prescreened for *MET*ex14 skipping in TBx and/or LBx samples, of whom 104 patients with confirmed *MET*ex14 skipping were screened for inclusion. A further 12 Asian patients entered screening directly via the LC-SCRUM program in Japan. Of the 116 screened patients, 106 patients in the combined biopsy group (TBx and/or LBx detection of *MET*ex14 skipping) were treated with tepotinib and had at least 18 months of follow-up, of which 48 patients had *MET*ex14 skipping detected by LBx, 83 patients were detected by TBx, and 25 patients were detected by both TBx and LBx. The Asian patients were from Japan (n = 38), South Korea (n = 20), Taiwan (n = 12), China (n = 30); and six Asian patients were enrolled from outside Asia (from The Netherlands [n = 1], Spain [n = 1], and the United States [n = 4]).

In the combined biopsy group of Asian patients, the median age was 70.5 years (range: 52–89), 39.6% were female, 43.4% had a history of smoking, 79.2% had adenocarcinoma, 73.6% had an ECOG PS of 1, and 47.2% of patients were treatment-naive (Table 1).

**Table 1:** Baseline characteristics.

Baseline characteristics		Asian population (N = 106)
Median age, years (range)		70.5 (52–89)
Sex, n (%)	Male	64 (60.4)
Smoking history,^a^ n (%)	Current smoker	0
	Former smoker	46 (43.4)
	Never smoker	58 (54.7)
Histology, n (%)	Adenocarcinoma	84 (79.2)
	Sarcomatoid	5 (4.7)
	Squamous	7 (6.6)
	Adenosquamous	3 (2.8)
	Large cell	1 (0.9)
	Other	6 (5.7)
ECOG PS, n (%)	0	28 (26.4)
	1	78 (73.6)
Presence of BM at baseline,^b^ n (%)	Yes	18 (17.0)
Line of therapy, n (%)	1L	50 (47.2)
	2L	36 (34.0)
	2L+	56 (52.8)
*MET*ex14 skipping detection, n (%)	T+	83 (78.3)
	L+	48 (45.3)
	T+ and L+	25 (23.6)

*1L* first line, *2L*, second line, 2L+, second or later line, *BM* brain metastases, *ECOG PS* Eastern Cooperative Oncology Group performance status, *L+* *MET*ex14 skipping detected in liquid biopsy, *MET*ex14 *MET* exon 14, *T+* *MET*ex14 skipping detected in tissue biopsy.

^a^Smoking history was missing in two patients.

^b^Identified at baseline (investigator or independent review).

### Efficacy in the overall Asian population (combined biopsy group)

**Fig. 1:**
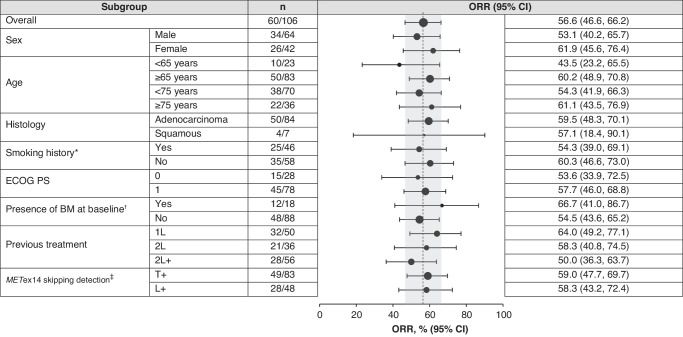
ORR in subgroups according to baseline characteristics. *Smoking history was missing in two patients. ^†^Identified at baseline (investigator or independent review). ^‡^Patients who tested positive by both methods are included in both sets; testing by both methods was not a requirement for study entry. 1L first line, 2L second line, 2L+ second or later line, BM brain metastases, CI confidence interval, ECOG PS Eastern Cooperative Oncology Group performance status; L+ *MET*ex14 skipping detected in liquid biopsy, *MET*ex14 *MET* exon 14, ORR objective response rate, T+ *MET*ex14 skipping detected in tissue biopsy.

**Fig. 2:**
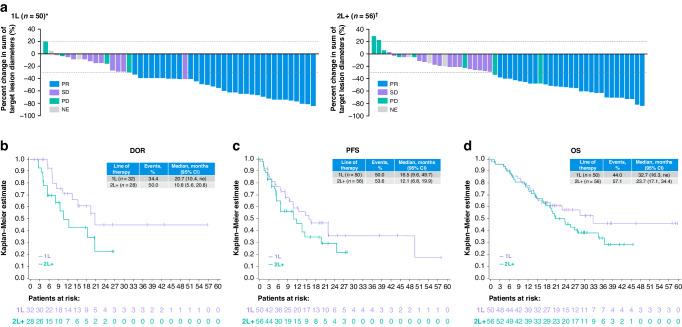
Efficacy outcomes in Asian patients according to line of therapy in the combined biopsy group (TBx and/or LBx detection of *MET*ex14 skipping). **a** Tumor response by independent review. **b** DOR by independent review. **c** PFS by independent review. **d** OS. *One patient is not shown due to baseline/on-treatment measurements not being available. ^†^Two patients are not shown due to baseline/on-treatment measurements not being available. 1L first line, 2L+ second or later line, CI confidence interval, DOR duration of response, LBx liquid biopsy, *MET*ex14 *MET* exon 14, NE not evaluable, ne not estimable, OS overall survival, PD progressive disease, PFS progression-free survival, PR partial response, SD stable disease, TBx tissue biopsy.

Among the 106 Asian patients in the combined biopsy group, the ORR across treatment lines was 56.6% (95% CI: 46.6, 66.2) according to the IRC. In treatment-naive patients, the ORR was 64.0% (95% CI: 49.2, 77.1) and in previously treated patients, the ORR was 50.0% (95% CI: 36.3, 63.7) (Table 2, Figs. 1, 2a). All the responses were PRs as determined by IRC. In treatment-naive patients, in addition to the 32 patients with PR, a further 11 patients had stable disease (SD) as best overall response, providing a disease control rate (DCR) of 86.0%. In previously treated patients, the DCR was 76.8% (which included 28 patients with PR and 15 patients with SD as best overall response). The ORR was relatively consistent regardless of baseline characteristics and was maintained in patients ≥75 years (ORR: 61.1% [95% CI: 43.5, 76.9]), and in patients with brain metastases at baseline (ORR: 66.7% [95% CI: 41.0, 86.7]) (Fig. 1).

**Table 2:** Efficacy of tepotinib in Asian patients based on IRC.

Efficacy (IRC)	Overall Asian patients			T+ Asian patients			L+ Asian patients		
	Combined (N = 106)	Treatment-naive (n = 50)	Previously treated (n = 56)	Combined (n = 83)	Treatment-naive (n = 42)	Previously treated (n = 41)	Combined (n = 48)	Treatment-naive (n = 21)	Previously treated (n = 27)
ORR, n, (%) (95% CI)	60 (56.6) (46.6, 66.2)	32 (64.0) (49.2, 77.1)	28 (50.0) (36.3, 63.7)	49 (59.0) (47.7, 69.7)	27 (64.3) (48.0, 78.4)	22 (53.7) (37.4, 69.3)	28 (58.3) (43.2, 72.4)	15 (71.4) (47.8, 88.7)	13 (48.1) (28.7, 68.1)
mDOR, months (95% CI)	18.5 (10.4, ne)	20.7 (10.4, ne)	10.8 (5.6, 20.8)	15.2 (8.3, ne)	19.4 (8.3, ne)	9.7 (5.6, ne)	18.5 (6.9, 20.7)	19.4 (6.9, ne)	10.8 (4.2, ne)
mPFS, months (95% CI)	13.8 (10.8, 22.0)	16.5 (9.6, 49.7)	12.1 (6.8, 19.9)	14.7 (10.8, 22.1)	15.9 (9.6, ne)	13.8 (6.9, ne)	11.0 (6.7, 19.9)	16.5 (6.9, ne)	6.9 (4.1, 13.8)
mOS, months (95% CI)	25.5 (19.3, 36.4)	32.7 (16.3, ne)	23.7 (17.1, 34.4)	27.8 (19.6, ne)	32.7 (19.1, ne)	25.5 (17.7, ne)	20.4 (14.2, 34.4)	28.5 (14.2, ne)	19.9 (10.9, 34.4)

*CI* confidence interval, *DOR* duration of response, *IRC* independent review committee, *L+* METex14 skipping detected in liquid biopsy, *m* median, *ne* not estimable, *ORR* objective response rate, *OS* overall survival, *PFS* progression-free survival, *T+* METex14 skipping detected in tissue biopsy.

Across treatment lines, in the combined biopsy group of the 106 Asian patients, the median DOR (mDOR) was 18.5 months (95% CI: 10.4, not estimable [ne]) by IRC (Supplementary Fig. 1). The mDOR was 20.7 (95% CI: 10.4, ne) in treatment-naive patients, and 10.8 months (95% CI: 5.6, 20.8) in previously treated patients (Table 2, Fig. 2b).

In the combined biopsy group, the mPFS by IRC was 13.8 months (95% CI: 10.8, 22.0) across treatment lines (Supplementary Fig. 1), 16.5 months (95% CI: 9.6, 49.7) in treatment-naive patients, and 12.1 months (95% CI: 6.8, 19.9) in previously treated patients (Table 2, Fig. 2c). The mOS was 25.5 months (95% CI: 19.3, 36.4) across treatment groups (Supplementary Fig. 1), 32.7 (95% CI: 16.3, ne) in treatment-naive patients, and 23.7 months (95% CI: 17.1, 34.4) in previously treated patients (Table 2, Fig. 2d).

At the time of data cut-off, in the overall Asian population of the combined biopsy group, tepotinib treatment was ongoing in 16 (15.1%) patients, while 90 (84.9%) patients had discontinued tepotinib. Out of the 16 patients whose tepotinib treatment was ongoing, 14 patients were in the treatment-naive group, and two patients were in the previously treated group. Out of the 90 patients who discontinued tepotinib, 37 (34.9%) patients received no subsequent anti-cancer therapy (due to death [24 patients, 22.6%], patient withdrawal of consent [seven patients, 6.6%], and other reasons [six patients, 5.7%]), while 53 (50.0%) patients received subsequent anti-cancer therapy, including cytotoxic therapy (32 [30.2%]), immunotherapy (23 [21.7%]), small molecules (12 [11.3%]), and monoclonal antibodies (9 [8.5%]). Twenty-six (24.5%) patients received a single line of subsequent anti-cancer therapy and 27 (25.5%) patients received two or more lines. Across all subsequent therapies, five (4.7%) patients had PR and 18 (17.0%) patients had SD as best response; the median longest DOR was 8.0 months and the median longest PFS was 4.0 months.

### Efficacy in the Asian population in TBx and LBx groups

In the Asian patients enrolled by TBx (T+; n = 83), the ORR was 59.0% (95% CI: 47.7, 69.7) across treatment lines, 64.3% (95% CI: 48.0, 78.4) in treatment-naive patients, and 53.7% (95% CI: 37.4, 69.3) in previously treated patients by IRC. In patients enrolled by LBx (L+; n = 48), the ORR was 58.3% (95% CI: 43.2, 72.4) across treatment lines, 71.4% (95% CI: 47.8, 88.7) in treatment-naive patients, and 48.1% (95% CI: 28.7, 68.1) in previously treated patients (Table 2).

In the T+ group, across treatment lines (n = 83), the mDOR was 15.2 months (95% CI: 8.3, ne), mPFS was 14.7 months (95% CI: 10.8, 22.1), and mOS was 27.8 months (95% CI: 19.6, ne) by IRC (Table 2**;** Supplementary Fig. 2). In the T+ group, in treatment-naive patients (n = 42), the mDOR was 19.4 (95% CI: 8.3, ne), mPFS was 15.9 months (95% CI: 9.6, ne), and mOS was 32.7 months (95% CI: 19.1, ne); in previously treated patients (n = 41), the mDOR was 9.7 months (95% CI: 5.6, ne), mPFS was 13.8 months (95% CI: 6.9, ne), and mOS was 25.5 months (95% CI: 17.7, ne) (Table 2).

In the L+ group, across treatment lines (n = 48), the mDOR was 18.5 months (95% CI: 6.9, 20.7), mPFS was 11.0 months (95% CI: 6.7, 19.9), and mOS was 20.4 months (95% CI: 14.2, 34.4) by IRC (Table 2**;** Supplementary Fig. 2). In the L+ group, in treatment-naive patients, the mDOR was 19.4 (95% CI: 6.9, ne), mPFS was 16.5 (95% CI: 6.9, ne), and mOS was 28.5 months (95% CI: 14.2, ne); in previously treated patients, the mDOR was 10.8 months (95% CI: 4.2, ne), mPFS was 6.9 months (95% CI: 4.1, 13.8), and mOS was 19.9 months (95% CI: 10.9, 34.4) (Table 2).

### HRQoL in the Asian population (combined biopsy group)

**Fig. 3:**
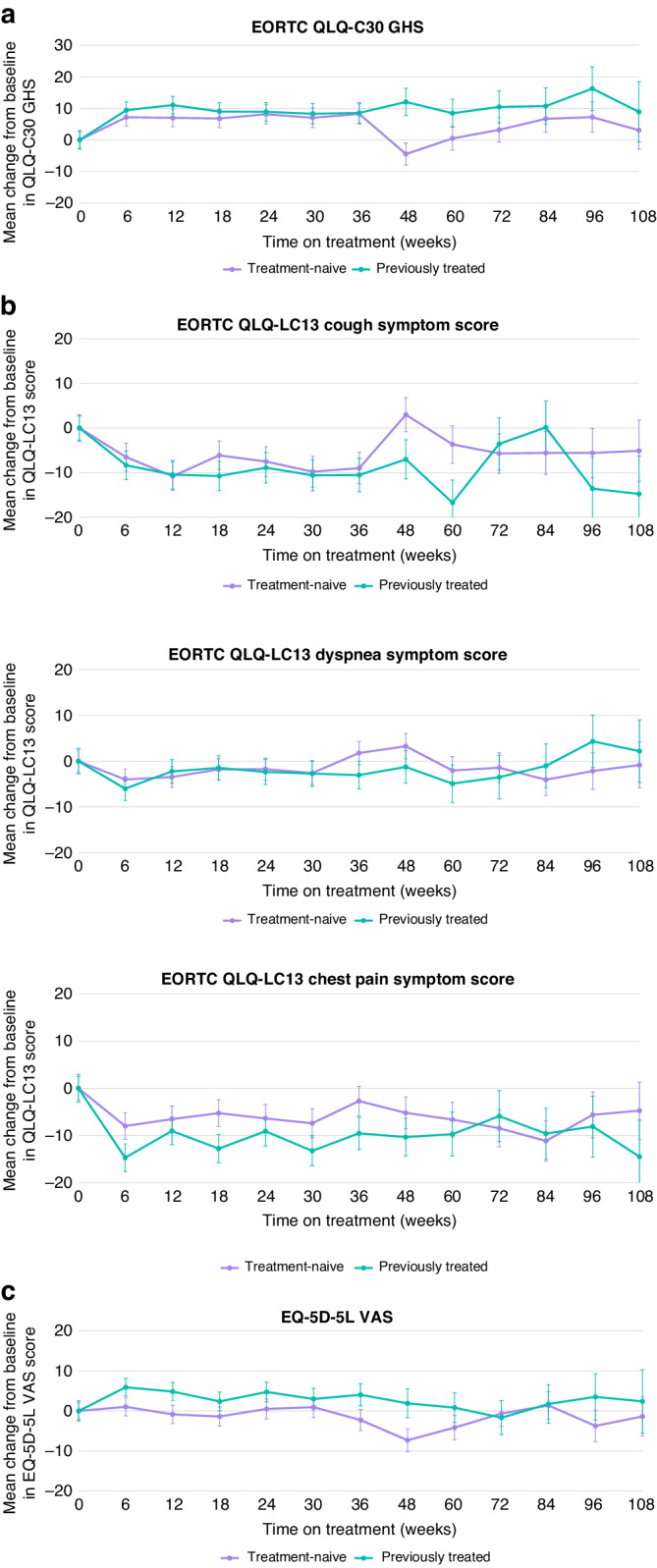
Mean change from baseline in PROs for treatment-naive and previously treated patients. **a** EORTC QLQ-C30 GHS. **b** EORTC QLQ-LC13 cough, dyspnea, and chest pain symptom scores. **c** EQ-5D-5L VAS scores. Error bars indicate SEs. EORTC European Organisation for the Research and Treatment of Cancer, EQ-5D-5L European Quality of Life five-dimension five-level; GHS, global health score, PRO patient-reported outcome, QLQ-C30 Quality of Life Questionnaire Core 30, QLQ-LC13 Quality of Life Questionnaire Lung Cancer 13, SE standard error, VAS visual analogue scale.

For the HRQoL analyses, only patients enrolled in Asia (China, Japan, South Korea, and Taiwan) were included. In the combined biopsy group of Asian patients, the number of patients who completed the EORTC QLQ-LC13 symptom score, EORTC QLQ-C30 GHS, and EQ-5D-5L visual analogue scale (VAS) were 100 patients overall (i.e. across treatments), of whom 48 patients were treatment-naive and 51 patients were previously treated; baseline PRO score observations were unavailable for one patient. Mean changes from baseline (standard error) in cough, chest pain and dyspnea as part of the EORTC QLQ-LC13 symptom score showed stability, with a numerical improvement in cough (−10.81 [3.17] in treatment-naive and −10.47 [3.21] in previously treated patients, at Week 12), dyspnea (−3.46 [2.30] in treatment-naive and −2.23 [2.59] in previously treated patients, at Week 12), and chest pain (−6.55 [2.81] in treatment-naive and −9.05 [2.93] in previously treated patients, at Week 12) (Fig. 3). Mean changes from baseline in EORTC QLQ-C30 GHS and functional scale scores, and EQ-5D-5L VAS scores demonstrated stability in patient quality of life over time. The mean scores for the EORTC QLQ-LC13 symptom score, EORTC QLQ-C30 GHS, and EQ-5D-5L VAS assessments for the overall Asian population, treatment-naive patients, and previously treated patients are shown in Supplementary Table 1 and time to deterioration for treatment-naive and previously treated patients is shown in Supplementary Fig. 3.

### Safety in the Asian population (combined biopsy group)

All-cause treatment-emergent AEs (TEAEs) and treatment-related AEs (TRAEs) in Asian patients are shown in Table 3. Among the 106 Asian patients in the safety population, TEAEs of any cause occurred in 105 (99.1%) patients, of whom 101 (95.3%) patients had TRAEs of any grade, with 42 (39.6%) patients having Grade 3 or higher TRAEs. The most common TRAEs (any grade [Grade ≥3]) were: peripheral edema (62.3% [7.5%]), creatinine increase (38.7% [0.9%]), diarrhea (32.1% [0.9%]), hypoalbuminemia (30.2% [3.8%]), alanine aminotransferase increase (ALT) (28.3% [2.8%]), and increased aspartate aminotransferase (AST) (22.6% [3.8%]). Decreased appetite, increased gamma-glutamyl transferase, anemia, and nausea were also common, but occurred in less than 20% of patients and were mostly mild (with Grade ≥3 occurring in less than 3% of patients).

**Table 3:** Tepotinib safety profile in Asian patients.

AEs, n (%)	Asian population (N = 106)	
	All-cause TEAEs	TRAEs
Any grade	105 (99.1)	101 (95.3)
Grade ≥3	65 (61.3)	42 (39.6)
Leading to dose reduction	35 (33.0)	32 (30.2)
Leading to permanent discontinuation	21 (19.8)	14 (13.2)
Leading to death	10 (9.4)	1 (0.9)
**TRAEs occurring in** ≥ **10% of patients, n (%)**	**Asian population (N** = **106)**	
	**Any grade**	**Grade** ≥ **3**^**a**^
Peripheral edema	66 (62.3)	8 (7.5)
Blood creatinine increase	41 (38.7)	1 (0.9)
Diarrhea	34 (32.1)	1 (0.9)
Hypoalbuminemia	32 (30.2)	4 (3.8)
ALT increase	30 (28.3)	3 (2.8)
AST increase	24 (22.6)	4 (3.8)
Decreased appetite	18 (17.0)	1 (0.9)
GGT increase	17 (16.0)	3 (2.8)
Anemia	16 (15.1)	3 (2.8)
Nausea	15 (14.2)	0
Amylase increase	13 (12.3)	1 (0.9)
Blood ALP increase	12 (11.3)	0
Lipase increase	12 (11.3)	5 (4.7)
Hyponatremia	11 (10.4)	2 (1.9)
White blood cell count decrease	11 (10.4)	0

*AEs* adverse events, *ALT* alanine aminotransferase, *ALP* alkaline phosphatase, *AST* aspartate aminotransferase, *CPK* creatine phosphokinase, *GGT* gamma-glutamyl transferase, *TEAEs* treatment-emergent AEs, *TRAEs* treatment-related adverse events.

^**a**^Four patients had Grade 4 TRAEs, including multiple organ dysfunction syndrome, ALT increase, AST increase, lymphocyte count decrease, and blood CPK increase (all n = 1); Grade 5 TRAEs were reported in one patient (multiple organ dysfunction syndrome).

In Asian patients, TRAEs led to a dose reduction in 32 (30.2%) patients and permanent treatment discontinuation in 14 (13.2%) patients. There were 10 deaths due to all-cause AEs and one death due to a TRAE of progressive disease or a lung cancer-related condition leading to multiple organ failure, which was considered treatment-related due to a missing causality report, as published previously [12]. The time on treatment in patients with dose reductions and/or interruptions is shown in Supplementary Fig. 4.

## Discussion

In VISION (Cohort A + C), tepotinib showed meaningful activity in Asian patients in both treatment-naive and previously treated patients, irrespective of *MET*ex14 skipping detection method. Efficacy was particularly durable in patients receiving tepotinib as first-line therapy with an ORR of 64.0% in the combined biopsy group (64.3% in T+ patients and 71.4% in L+ patients) by IRC. The results reported here comprise the largest population of Asian patients with *MET*ex14 skipping NSCLC in a MET inhibitor trial to date.

These data from Asian patients in VISION support the use of tepotinib in first or subsequent lines of therapy, in line with a recently published Asian Thoracic Oncology Research Group (ATORG) consensus which recommends considering tepotinib in first, second, or subsequent lines of therapy in patients with *MET*ex14 skipping metastatic NSCLC [15]. Results from the VISION study reported here were based on TBx assay testing performed on RNA. The ATORG consensus recommendations recognize that the use of RNA-based TBx may be more accurate than DNA-based TBx for detection of *MET*ex14 skipping, and that the use of LBx (ctDNA in plasma) is increasingly being adopted [15]. LBx offers the advantage of being more practical, convenient, accessible, and less invasive than TBx; while extracting high quality RNA from TBx can be challenging [8, 15, 16]. Analysis of *MET* alterations in LBx (ctDNA in plasma) can be used as an adjunctive approach, and can be particularly useful when TBx samples are inadequate or unavailable [8, 15, 16]. In addition, ctDNA dynamics in consecutive LBx samples may be used to monitor response and progression to treatment in NSCLC [8, 10, 17, 18]. In the VISION study, the Asian patients’ results are similar to the global study population, which show concordance in results for identifying patients and predicting treatment outcomes in treatment-naive and previously treated patients with confirmed *MET*ex14 skipping NSCLC, using LBx or TBx [10]. While ORR and DOR in Asian patients was generally similar between the T+ and L+ groups, the L+ subgroup showed trends for shorter median PFS in previously treated patients and shorter median OS irrespective of prior treatment. Corresponding trends were seen in the overall population and likely reflect a poorer prognosis of the L+ subgroup due to their greater baseline disease burden [19]. Given the correlation between tumor size and ctDNA shedding [20], LBx may preferentially identify patients with higher tumor load. Overall, tepotinib showed meaningful activity in patients enrolled by TBx or LBx, demonstrating that both methods are appropriate for identifying patients likely to benefit with tepotinib.

Similar to the global overall population in VISION, subgroup data show consistent efficacy in Asian patients regardless of baseline characteristics, including those with baseline brain metastases (n = 18; ORR 66.7%) [10]. These intracranial efficacy results support the use of MET inhibitors in this population.

Besides tepotinib, capmatinib and savolitinib (approved in China in previously treated patients) are included in the ATORG consensus recommendations [15]. Apart from these MET inhibitors, gumarontinib also has demonstrated efficacy in Asian patients with *MET*ex14 skipping NSCLC [21–23]. In the Asian subgroup analysis of the GEOMETRY mono-1 study evaluating capmatinib in patients with *MET*ex14 skipping NSCLC (n = 20), the overall response rate was 45% (95% CI: 23, 69) by IRC regardless of line of therapy, 67% (95% CI: 9, 99) in treatment-naive patients (n = 3) and 41% (95% CI: 18, 67) in previously treated patients (n = 17); in the treatment-naive cohort, two (66.7%) patients had PR and one (33.3%) patient had SD, while in the previously treated cohort, seven (41.2%) and five (29.4%) patients had PR and SD, respectively [21]. In the savolitinib study in Chinese patients with *MET*ex14 skipping NSCLC, at a median follow-up of 17.6 months, savolitinib was found to have an IRC-assessed ORR of 42.9% (95% CI: 31.3, 55.3; 30 of 70 patients), mPFS of 6.8 months (95% CI: 4.2, 9.6), and mOS of 12.5 months (95% CI: 10.5, 23.6) [24]. In treatment-naive patients (n = 28), the ORR was 46.4% (95% CI: 27.5, 66.1) and mPFS was 5.6 months (95% CI: 4.1, 9.6); in previously treated patients (n = 42), the ORR was 40.5% (95% CI: 25.6, 56.7) and mPFS was 6.9 months (95% CI: 4.1, 19.3) [24]. In the GLORY study that investigated gumarontinib in patients with *MET*ex14 skipping NSCLC, the ORR was 66% (95% CI: 54, 76) in the overall population (n = 79), 71% (95% CI: 55.0, 83.0) in treatment-naive patients (n = 44), and 60% (95% CI: 42, 76) in previously treated patients (n = 35); the overall mDOR and mPFS were 8.3 months (95% CI: 6.3, ne) and 8.5 months (95% CI: 7.6, 9.7), respectively [23]. The efficacy results for Asian patients receiving tepotinib in the VISION study, across treatment lines in treatment-naive and previously treated patients, compare favorably with the aforementioned data of other MET inhibitors, supporting the use of MET inhibitors in this patient population.

In the VISION study, HRQoL remained stable during treatment in Asian patients. This is the only study to report HRQoL in Asian patients with *MET*ex14 skipping NSCLC treated with a MET inhibitor.

Tepotinib was generally well tolerated in Asian patients, with a low proportion of TRAEs leading to discontinuation. The most common TRAEs in the Asian population in the VISION study were peripheral edema, creatinine increase, and diarrhea. There was no evidence that creatinine increase was associated with renal impairment [25, 26]; a potential explanation is that serum creatinine is increased by inhibition of renal transporters [26–30]. Incidence of creatinine increase appeared to be more common in Asian patients than in White patients in VISION [25]. In the other three studies with MET inhibitors in patients with *MET*ex14 skipping NSCLC, the most common TRAEs were: peripheral edema, nausea, vomiting, and increased blood creatinine (GEOMETRY mono-1, capmatinib); peripheral edema, nausea, increased ALT, increased AST, and increased blood creatinine (savolitinib study in Chinese patients); and edema, hypoalbuminemia, headache, loss of appetite, nausea, increased blood bilirubin, increased ALT, vomiting, and increased AST (GLORY, gumarontinib) [22, 23, 30]. Although cross-trial safety comparisons must be drawn with caution, the incidence of Grade 3 or higher TRAEs was potentially lower with tepotinib in Asian patients (39.6%) compared with the overall populations of the savolitinib (45.7%), gumarontinib (53.6%), or capmatinib (45.7%) trials in patients with *MET*ex14 skipping [22, 23, 30]. The most common TRAE in all four studies was peripheral edema, a MET inhibitor class effect, which was reported in 62.3%, 55.7%, 73.8%, and 50.3%, respectively [22, 23, 30]. Nausea and vomiting appeared less frequent, while diarrhea appeared more common, with tepotinib than with the other MET inhibitors [22, 23, 30].

Prevalence of *MET*ex14 skipping in Western patients is slightly higher than in Asian patients (median 3.4% vs 1.7%) [15]; in the two largest *MET* alteration analyses in lung cancer populations published to date, *MET*ex14 was identified less frequently in China (175 of 18,112 [1.1%] patients) compared with the United States (298 of 11,205 [2.7%] patients) [31, 32]. Detection rates in VISION may be biased due to trial-related inclusion/exclusion criteria.

VISION is the largest clinical trial to date in patients with *MET*ex14 skipping. Given the poor prognosis [33], limited efficacy of standard of care therapies in these patients [34], and the rarity of the alteration [35], the durable clinical benefit shown by tepotinib in this single-arm trial has provided strong evidence to support its approval in many countries globally, including several in Asia. In countries such as the US, where tepotinib was granted conditional approval based on initial results from VISION, subsequent data from the study is being used to fulfill post-marketing requirements by providing more mature results in a larger patient cohort [12]. Further evidence to support clinical use of tepotinib in this population are expected from the ongoing MOMENT registry [36].

A limitation of the VISION study is its design; as a Phase II, open-label, non-randomized study, there is no direct comparison to other MET inhibitors or existing agents. However, the feasibility of recruiting enough patients with confirmed *MET*ex14 skipping NSCLC (using LBx or TBx) in a randomized controlled clinical trial in this setting is also limited. Lastly, selection bias might have been introduced in samples submitted for central biomarker testing in the minority of patients who were initially positive for *MET*ex14 skipping by local testing.

In conclusion, in the VISION study, tepotinib demonstrated robust and durable clinical activity, particularly as a first-line treatment, with stability in HRQoL and a manageable safety profile in Asian patients with *MET*ex14 skipping NSCLC. Overall, data from VISION support the use of tepotinib in Asian patients with *MET*ex14 skipping NSCLC, in first or subsequent lines of therapy.

### Supplementary information

Supplementary materials PDF

## Data Availability

Any requests for data by qualified scientific and medical researchers for legitimate research purposes will be subject to Merck’s (CrossRef Funder ID: 10.13039/100009945) Data Sharing Policy. All requests should be submitted in writing to Merck’s data sharing portal (https://www.merckgroup.com/en/research/our-approach-to-research-and-development/healthcare/clinical-trials/commitment-responsible-data-sharing.html). When Merck has a co-research, co-development, or co-marketing or co-promotion agreement, or when the product has been out-licensed, the responsibility for disclosure might be dependent on the agreement between parties. Under these circumstances, Merck will endeavor to gain agreement to share data in response to requests.
