# Supplementary material for: Efficacy and safety of tepotinib in Asian patients with advanced NSCLC with MET exon 14 skipping enrolled in VISION
Source: Br J Cancer. 2024 Apr 4;130(10):1679–86. doi: 10.1038/s41416-024-02615-9 (PMC11091176; doi:10.1038/s41416-024-02615-9)
Supplement: Supplementary file 1 — Supplementary materials PDF [file 41416_2024_2615_MOESM1_ESM.pdf]

Supplementary Table 1. PROs mean change from baseline

PRO, mean (SE)		Overall (N=99)[§]	Treatment-naïve (n=48)	Previously treated (n=51)
EORTC QLQ-C30 GHS*		3.85 (1.83)	2.71 (2.63)	4.94 (2.54)
EORTC QLQ-LC13 symptom score[†]	Cough	−6.93 (1.76)	−5.94 (2.54)	−7.89 (2.43)
	Dyspnea	−1.62 (1.44)	−1.46 (1.69)	−1.80 (2.32)
	Chest pain	−6.72 (1.71)	−5.26 (1.91)	−8.10 (2.78)
EQ-5D-5L VAS[‡]		−0.98 (1.46)	−3.28 (1.82)	1.28 (2.22)

An increase or decrease of >10 points was considered to be clinically meaningful.
*EORTC QLQ-C30 GHS patient functioning scales – higher scores indicate greater function (scale 0–100). [†]EORTC QLQ-LC13 symptom score – lower scores indicate milder symptoms (scale 0–100). [‡]EQ-5D-5L VAS – higher scores indicate greater function (scale 0–100). [§]There were 100 Asian patients in total; however, there were no baseline PRO score observations for one patient.
EORTC, European Organisation for the Research and Treatment of Cancer; EQ-5D-5L, European Quality of Life five-dimension five-level; GHS, global health score; PRO, patient-reported outcome; QLQ-C30, Quality of Life Questionnaire Core 30; QLQ-LC13, Quality of Life Questionnaire Lung Cancer 13; SE, standard error; VAS, visual analogue scale.

Supplementary Figure 1. DOR, PFS, and OS in overall population, by independent review, in the combined biopsy group (TBx and/or LBx detection of *MET*ex14 skipping)

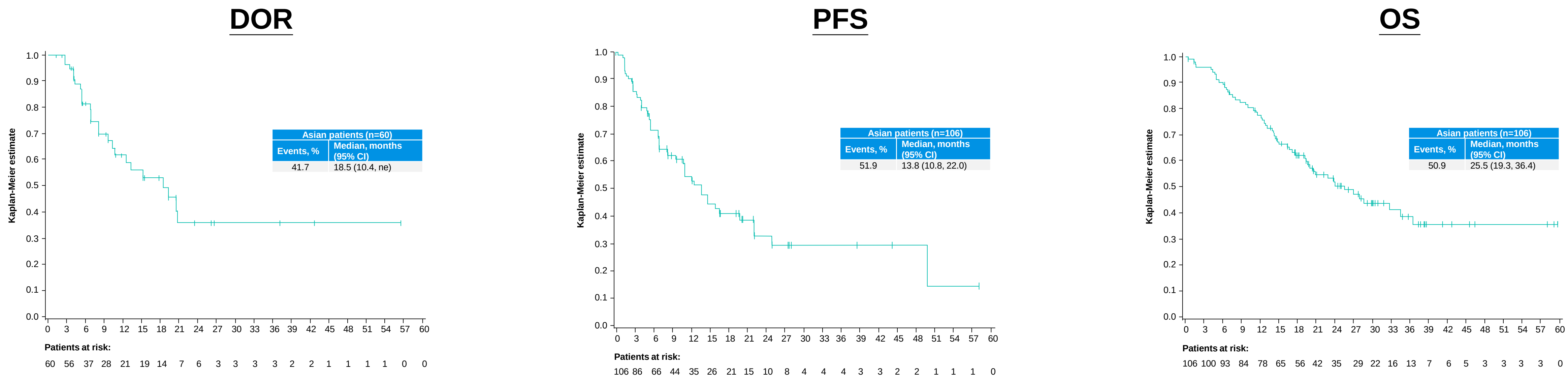

CI, confidence interval; DOR, duration of response; LBx, liquid biopsy; *MET*ex14, *MET* exon 14; ne, not estimable; OS, overall survival; PFS, progression-free survival; TBx, tissue biopsy.

Supplementary Figure 2. DOR, PFS, and OS in T+ and L+ patients

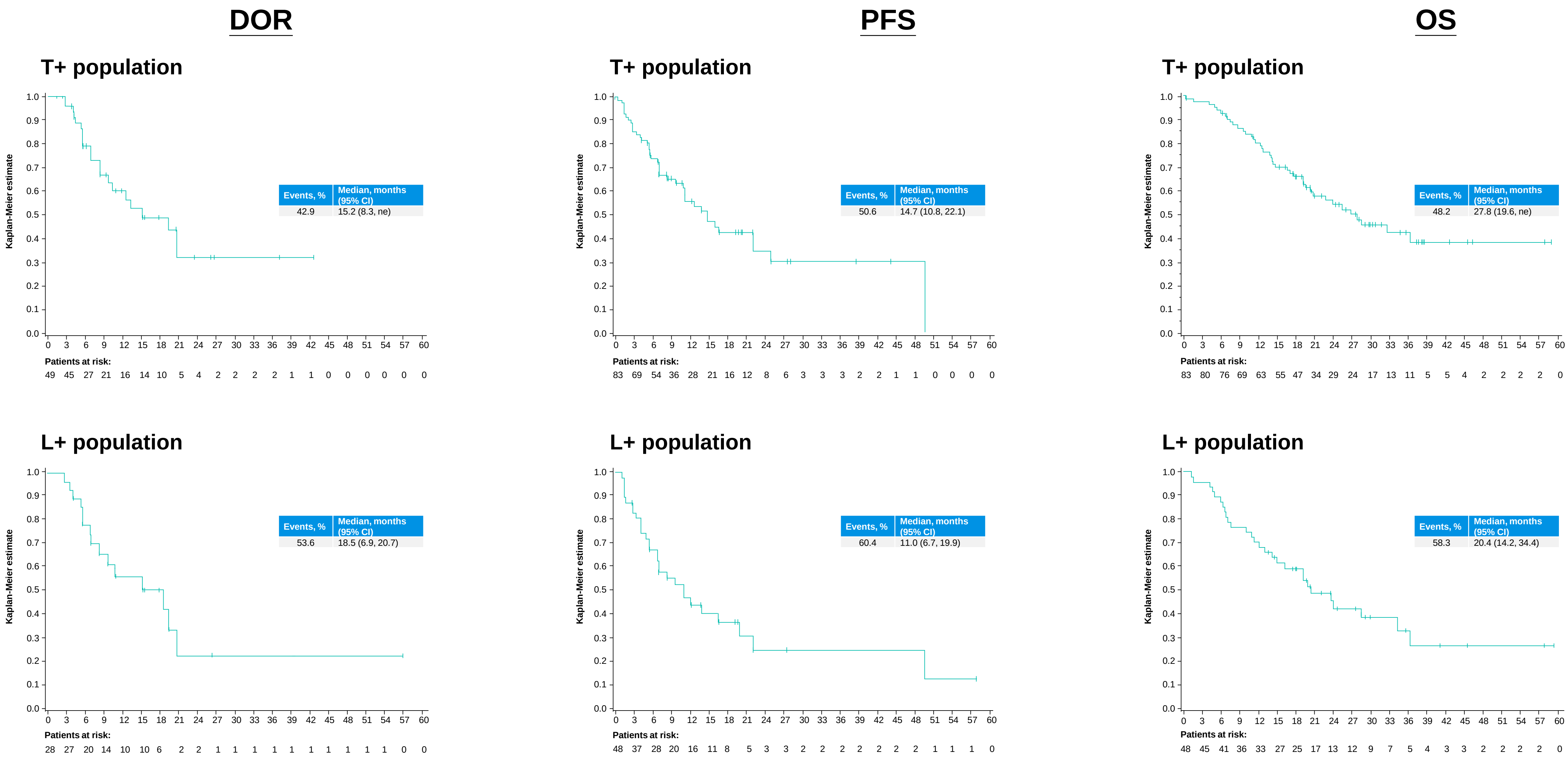

CI, confidence interval; DOR, duration of response; ne, not estimable; L+, *MET*ex14 skipping detected in liquid biopsy; *MET*ex14, *MET* exon 14; OS, overall survival; PFS, progression-free survival; T+, *MET*ex14 skipping detected in tissue biopsy.

Supplementary Figure 3. Time to deterioration in (a) EORTC QLQ-C30 GHS, (b) EORTC QLQ-LC13 cough, dyspnea, and chest pain symptom scores, and (c) EQ-5D-5L VAS scores, for treatment-naïve and previously treated patients. Shaded areas indicate 95% CIs.

(a) EORTC QLQ-C30 GHS

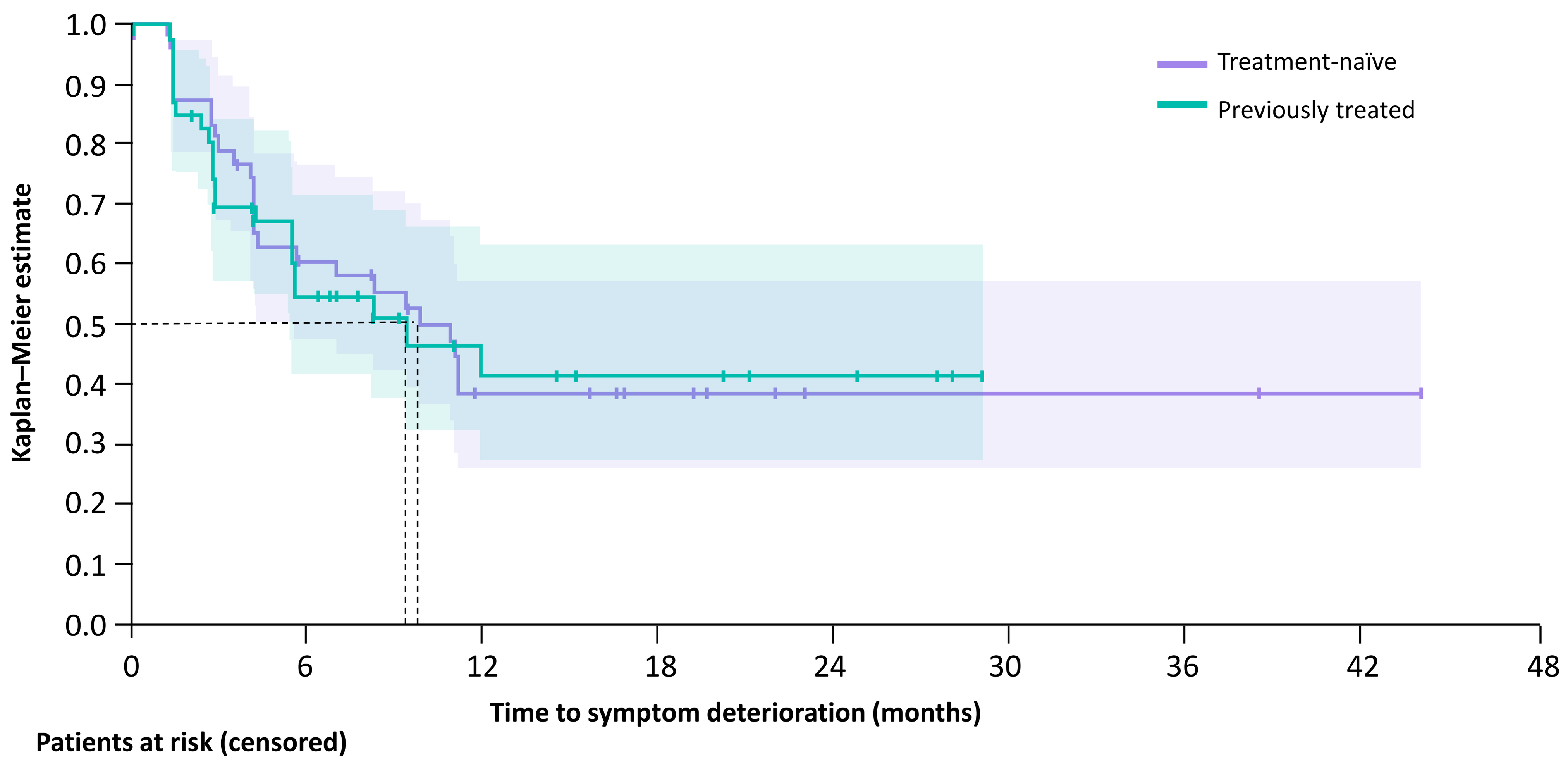

Patients at risk (censored)

	0	6	12	18	24	30	36	42
Treatment-naïve	48 (0)	24 (6)	12 (10)	7 (15)	2 (20)	2 (20)	2 (20)	1 (21)
Previously treated	52 (0)	21 (11)	8 (21)	6 (23)	4 (25)	0 (29)		

(b) EORTC QLQ-LC13 cough, dyspnea, and chest pain symptom scores

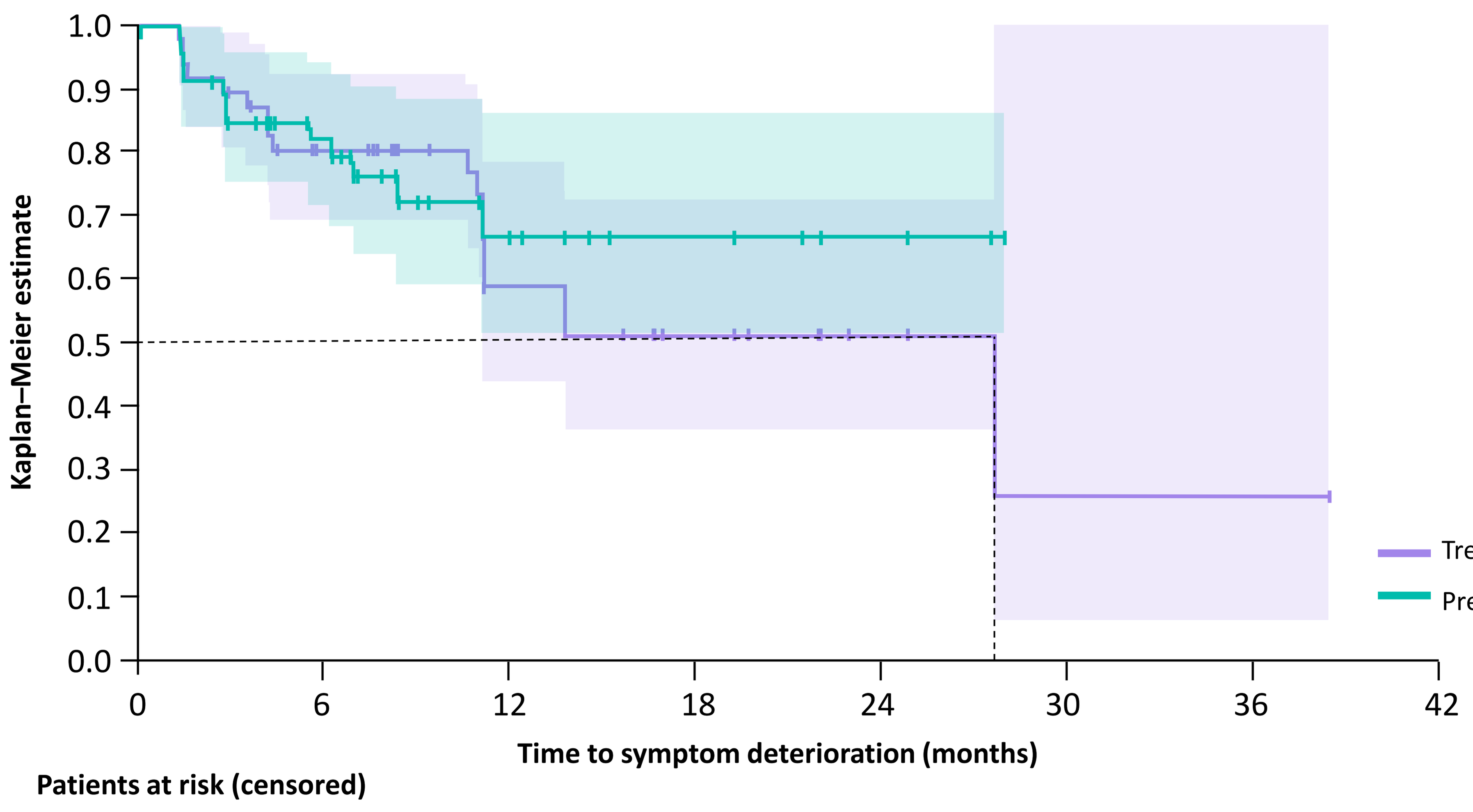

Patients at risk (censored)

	0	6	12	18	24	30	36
Treatment-naïve	48 (0)	32 (7)	15 (18)	8 (23)	3 (28)	1 (29)	1 (29)
Previously treated	52 (0)	30 (14)	11 (29)	6 (34)	3 (37)	0 (40)	

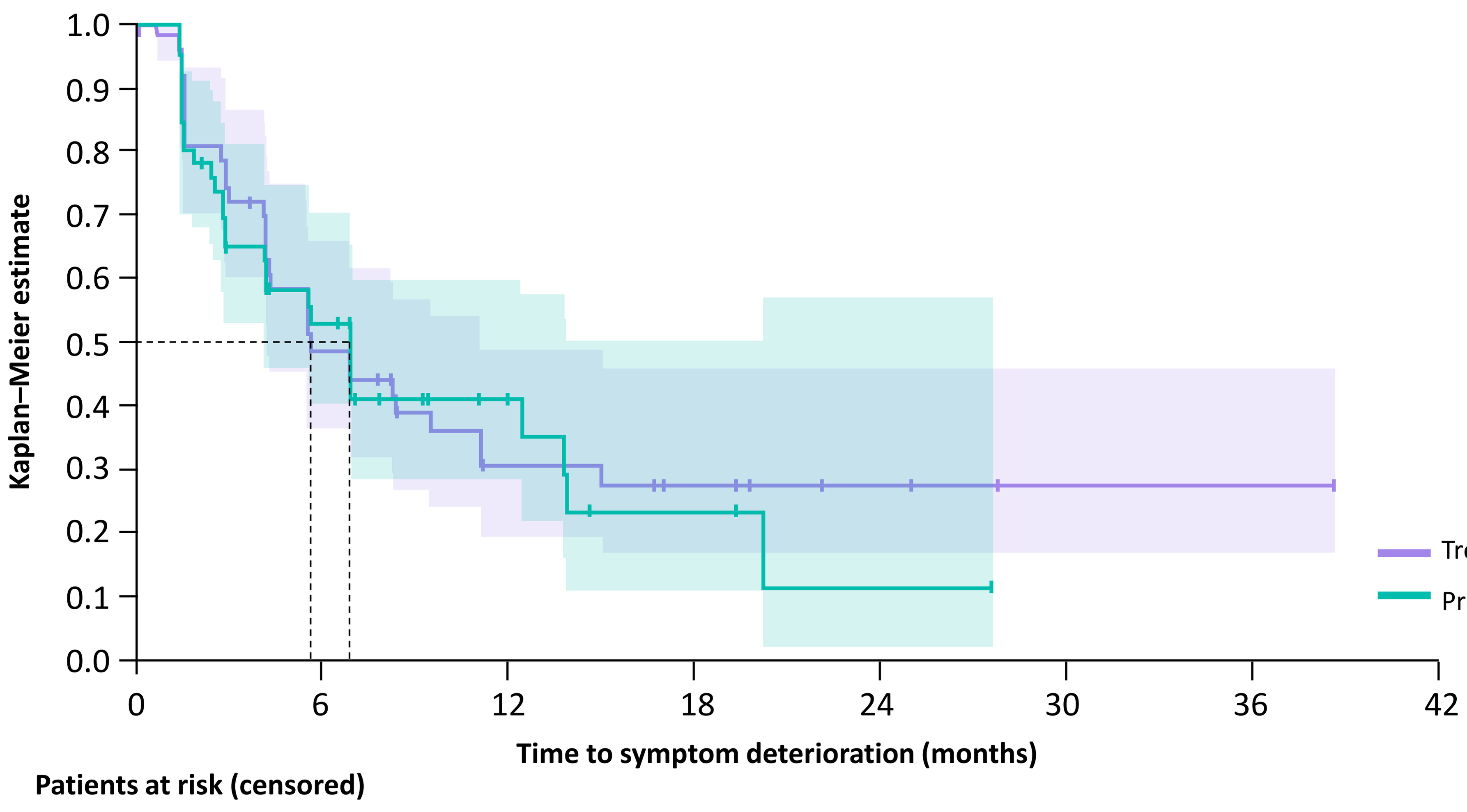

Patients at risk (censored)

	0	6	12	18	24	30	36
Treatment-naïve	48 (0)	21 (4)	10 (8)	7 (10)	3 (14)	1 (16)	1 (16)
Previously treated	52 (0)	20 (11)	7 (20)	3 (21)	1 (22)	0 (23)	

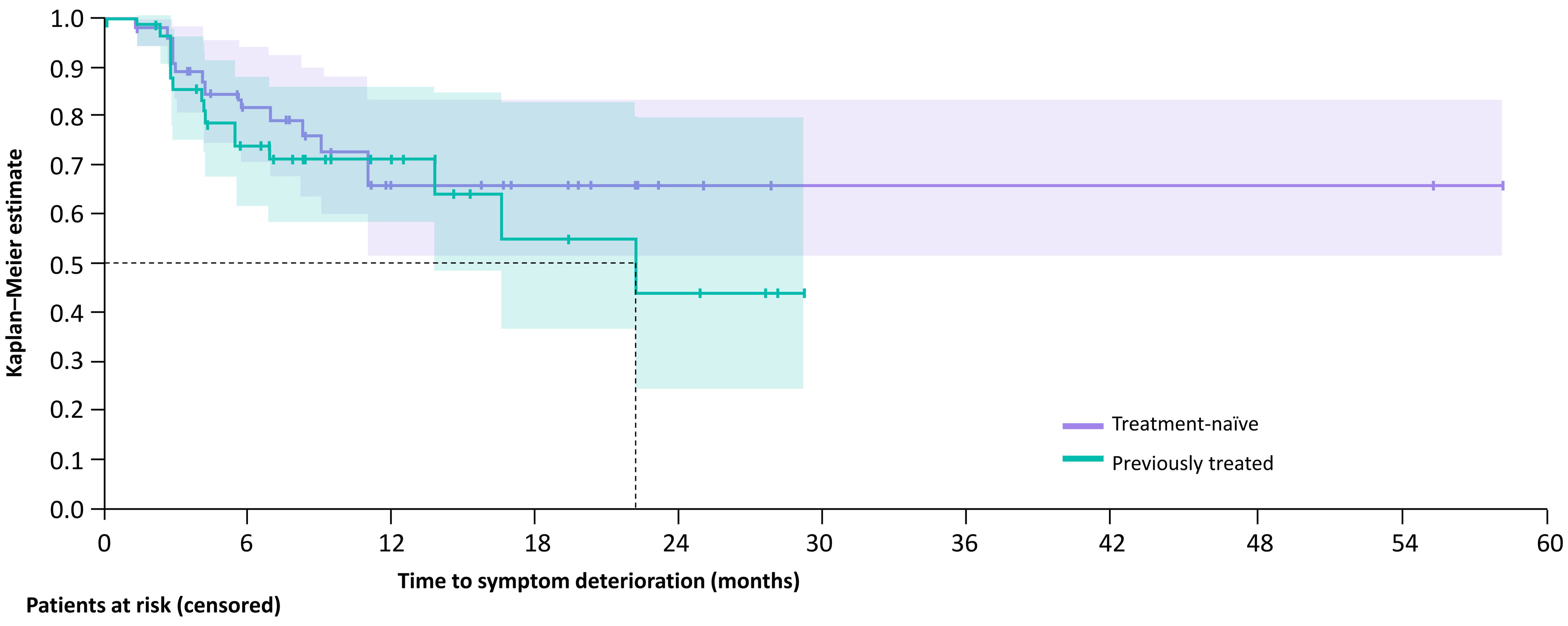

Patients at risk (censored)

	0	6	12	18	24	30	36	42	48	54	60
Treatment-naïve	48 (0)	30 (10)	15 (20)	11 (24)	4 (31)	2 (33)	2 (33)	2 (33)	2 (33)	2 (33)	0 (35)
Previously treated	52 (0)	30 (10)	14 (25)	6 (31)	4 (32)	0 (36)					

(c) EQ-5D-5L VAS

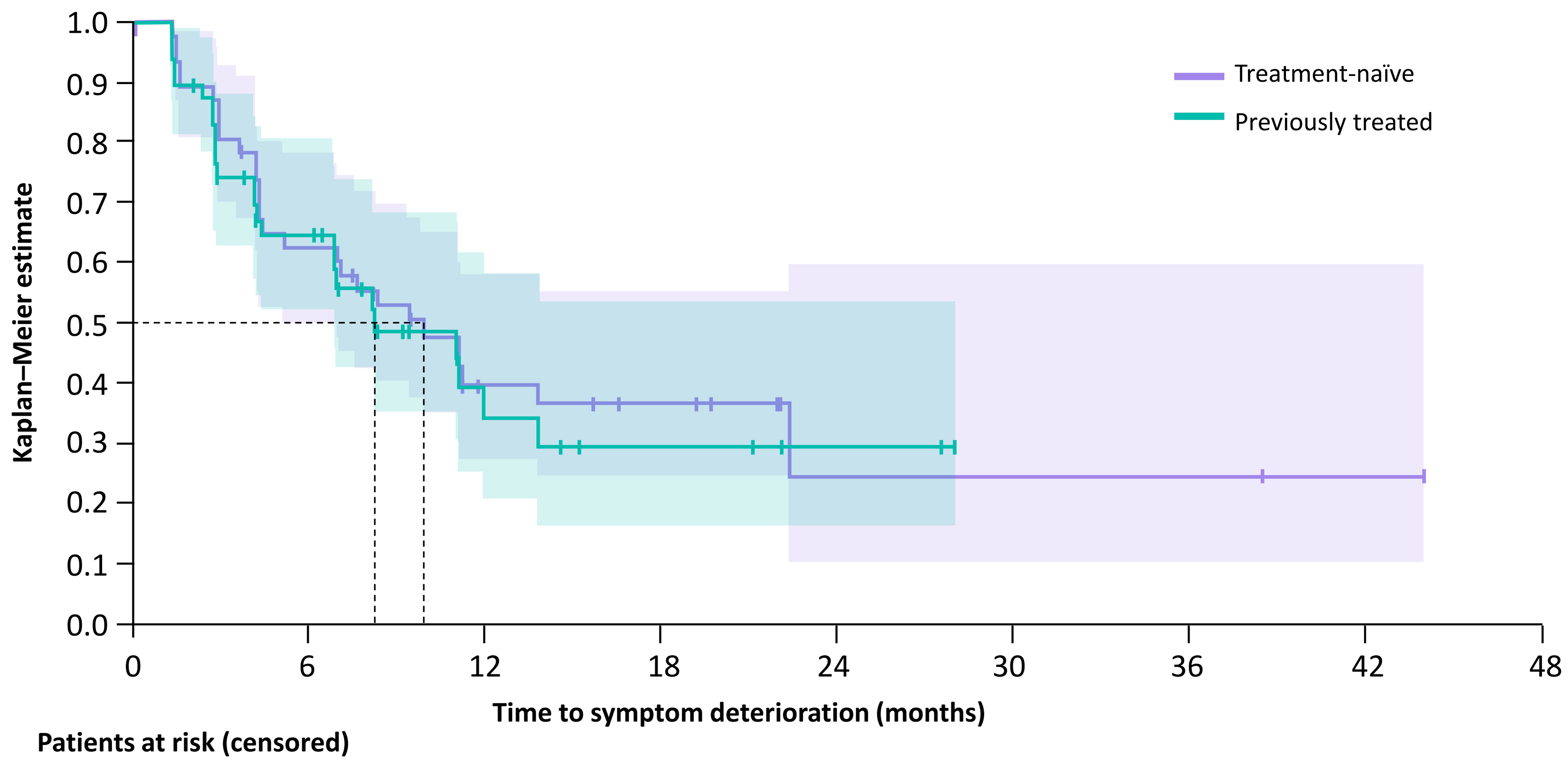

Patients at risk (censored)

	0	6	12	18	24	30	36	42
Treatment-naïve	48 (0)	27 (4)	12 (10)	8 (13)	2 (18)	2 (18)	2 (18)	1 (19)
Previously treated	52 (0)	26 (10)	7 (21)	4 (23)	2 (25)	0 (27)		

CI, confidence interval; EORTC, European Organisation for the Research and Treatment of Cancer; EQ-5D-5L, European Quality of Life five-dimension five-level; GHS, global health score; QLQ-C30, Quality of Life Questionnaire Core 30; QLQ-LC13, Quality of Life Questionnaire Lung Cancer 13; VAS, visual analogue scale.

Supplementary Figure 4. Time on treatment in patients with dose reductions and/or interruptions (n=59)

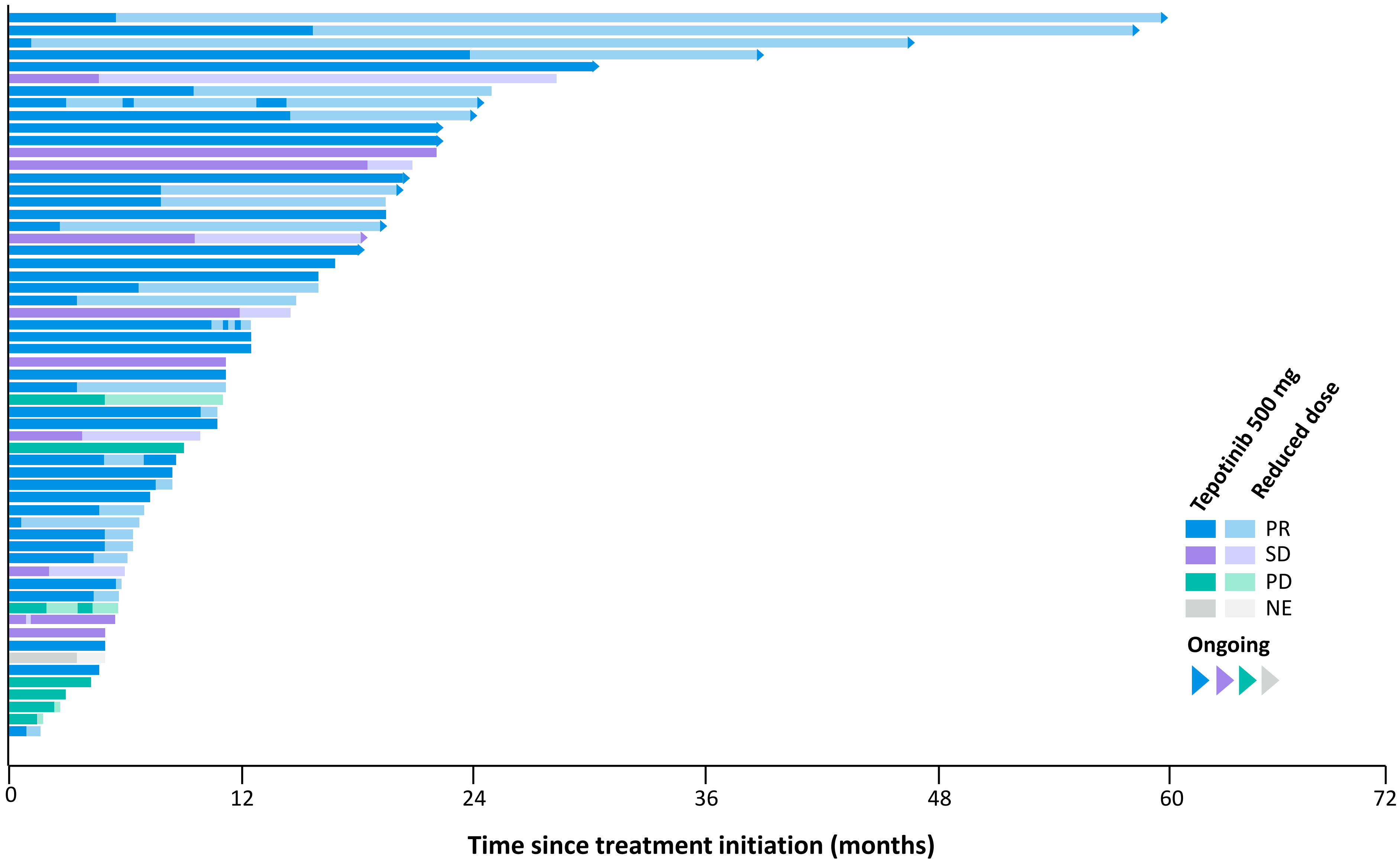

Patients indicated with single-color lines had only interruptions with no dose reductions, and all other patients had both treatment interruptions and dose reductions.

NE, not evaluable; PD, progressive disease; PR, partial response; SD, stable disease.
